# Association between *HOX* Transcript Antisense RNA Single-Nucleotide Variants and Recurrent Implantation Failure

**DOI:** 10.3390/ijms22063021

**Published:** 2021-03-16

**Authors:** Jeong Yong Lee, Eun Hee Ahn, Hyeon Woo Park, Ji Hyang Kim, Young Ran Kim, Woo Sik Lee, Nam Keun Kim

**Affiliations:** 1 Department of Biomedical Science, College of Life Science, CHA University, Seongnam 13488, Korea; smilee3625@naver.com (J.Y.L.); aabb1114@naver.com (H.W.P.); 2 CHA Bundang Medical Center, Department of Obstetrics and Gynecology, School of Medicine, CHA University, Seongnam 13496, Korea; bestob@chamc.co.kr (E.H.A.); bin0902@chamc.co.kr (J.H.K.); happyimam@naver.com (Y.R.K.); 3 CHA Gangnam Medical Center, Department of Obstetrics and Gynecology, School of Medicine, CHA University, Seoul 06135, Korea; wooslee@cha.ac.kr

**Keywords:** RIF, pregnancy, long non-coding RNA, single nucleotide variant, implantation

## Abstract

Recurrent implantation failure (RIF) refers to the occurrence of more than two failed in vitro fertilization–embryo transfers (IVF-ETs) in the same individual. RIF can occur for many reasons, including embryo characteristics, immunological factors, and coagulation factors. Genetics can also contribute to RIF, with some single-nucleotide variants (SNVs) reported to be associated with RIF occurrence. We examined SNVs in a long non-coding RNA, homeobox (*HOX*) transcript antisense RNA (*HOTAIR*), which is known to affect cancer development. *HOTAIR* regulates epigenetic outcomes through histone modifications and chromatin remodeling. We recruited 155 female RIF patients and 330 healthy controls, and genotyped *HOTAIR* SNVs, including rs4759314, rs920778, rs7958904, and rs1899663, in all participants. Differences in these SNVs were compared between the patient and control groups. We identified significant differences in the occurrence of heterozygous genotypes and the dominant expression model for the rs1899663 and rs7958904 SNVs between RIF patients and control subjects. These *HOTAIR* variants were associated with serum hemoglobin (Hgb), luteinizing hormone (LH), total cholesterol (T. chol), and blood urea nitrogen (BUN) levels, as assessed by analysis of variance (ANOVA). We analyzed the four *HOTAIR* SNVs and found significant differences in haplotype patterns between RIF patients and healthy controls. The results of this study showed that *HOTAIR* is not only associated with the development of cancer but also with pregnancy-associated diseases. This study represents the first report showing that *HOTAIR* is correlated with RIF.

## 1. Introduction

Recurrent implantation failure (RIF) refers to repeated embryo implantation failure in the same individual, which is associated with many potential causes [1,2,3]. Researchers generally refer to RIF as infertility, which is also associated with the repeated failure of in vitro fertilization–embryo transfer (IVF-ET). Many underlying causes of RIF have been reported, such as embryo characteristics, immunological factors, uterine features, coagulation factors, and genetics [2].

Long, non-coding RNAs (lncRNAs) refer to transcribed RNAs that are longer than 200 nt without an open reading frame (ORF). The functions of lncRNAs remain unclear, but lncRNAs participate in various roles, such as the regulation of gene expression, post-transcriptional modifications, and translation [4]. Various lncRNAs are associated with disease states, especially cancer occurrence [5,6,7]. However, the contributions of lncRNAs to pregnancy-associated complications, such as pre-eclampsia and recurrent pregnancy loss, have rarely been reported [8,9,10]

Homeobox (*HOX*) transcript antisense RNA (*HOTAIR*) is an lncRNA, located on chromosome 12q13.13 and encoded in the *HOXC* gene cluster [11], which consists of 6232 nucleotides [12]. *HOTAIR* recruits polycomb repressive complex 2 (PRC2), which initiates chromatin remodeling to induce a heterochromatin state, activating *HOXD* transcription by decreasing the trimethylation of histone H3K27 [12]. *HOTAIR* is also known to interact with the lysine-specific histone demethylate 1A (LSD1), which regulates histone H3K4 during epigenetic regulation [12]. *HOTAIR* has been reported to be a key regulator of cancer, including colorectal, prostate, gastric, and ovarian cancers [11,13]. However, whether *HOTAIR* contributes to pregnancy disorders remains unknown.

In this study, we investigated the occurrence of *HOTAIR* single nucleotide variants (SNVs) associated with changes in the risk of RIF. Single nucleotide variants (SNVs) have been associated with many diseases [5,14,15,16]. RIF has previously been associated with SNVs, and many studies have been published exploring these associations [14,17,18,19]. The occurrence of *HOTAIR* SNVs has also been reported in association with various diseases, including psoriasis, pre-eclampsia, and various cancers [13,20,21,22,23]. However, no studies have examined the associations between RIF and *HOTAIR* SNVs. To reveal the relationship between RIF and *HOTAIR* SNVs, we assessed the differences between RIF patients and healthy controls, by examining known *HOTAIR* gene polymorphisms, including rs4759314, rs920778, rs7958904, and rs1899663.

## 2. Materials and Methods

### 2.1. Study Population

Blood samples were obtained from 155 females with RIF and 330 healthy female controls. All study samples were collected from the Department of Obstetrics and Gynecology of CHA Bundang Medical Center (Seongnam, South Korea), between March 2010 and December 2012. The Institutional Review Board of CHA Bundang Medical Center reviewed and approved the study on 23 February 2010 (reference no. CHAMC2009-12-120). Informed consent was obtained from all participants. We defined RIF as the failure to achieve pregnancy following the completion of two fresh IVF-ET cycles, using >10 cleaved embryos, and serum human chorionic gonadotrophin concentrations of <5 U/mL, 14 days after ET. Individuals diagnosed with RIF due to anatomical, chromosomal, hormonal, infectious, autoimmune, or thrombotic causes were excluded from the study. Anatomical abnormalities were evaluated using several imaging modalities, including sonography, hysterosalpingogram, hysteroscopy, computed tomography, and magnetic resonance imaging. Karyotyping was performed using standard protocols to assess chromosomal abnormalities. We excluded hormonal causes of RIF, including hyperprolactinemia, luteal insufficiency, and thyroid disease, by measuring the concentrations of prolactin (PRL), thyroid-stimulating hormone (TSH), free thyroxine, follicle-stimulating hormone (FSH), LH, estradiol (E2), and progesterone in peripheral blood samples. To exclude lupus and antiphospholipid syndrome as potential autoimmune causes of RIF, we examined the levels of lupus anticoagulant and anticardiolipin antibodies, according to the protocols described in a previous study [24]. We evaluated thrombophilia by testing for protein C and S deficiencies and the presence of anti-α2 glycoprotein antibodies, using the methods described in a previous study [25]. All control participants had regular menstrual cycles, normal karyotype (46XX), and no history of pregnancy disease such as pregnancy loss or pre-eclampsia and at least one natural birth with healthy conditions.

### 2.2. Genotype Analysis

Genomic DNA was extracted from whole-blood samples, using the G-DEX II Genomic DNA Extraction kit (Intron Biotechnology Inc., Seongnam, Korea). DNA was diluted to 100 ng/µL with 1× Tris-EDTA (TE) buffer, and then 1 µL of each sample was used to amplify the polymorphisms.

All PCR experiments were performed using an AccuPower HotStart PCR PreMix (Bioneer Corporation, Daejeon, Korea). For the genotyping analysis, rs7958904 and rs920778 were analyzed using a Taq-man genotyping assay (Applied Biosystems, Foster City, CA, USA), whereas rs1899663 and rs4759314 genotyping was performed using polymerase chain reaction-restriction fragment length polymorphism (PCR-RFLP) analysis. Information regarding the primers and restriction enzymes used for PCR-RFLP are presented in Supplementary Table S1. Taq-man probes were obtained directly from Applied Biosystems, and genotyping was performed using the manufacturer’s protocols.

### 2.3. Assessment of Blood Coagulation Status

We measured the platelet count (PLT), white blood cells (WBCs), and hemoglobin (Hgb) levels using the Sysmex XE 2100 Automated Hematology System (Sysmex Corporation, Kobe, Japan). We used the ACL TOP automated photo-optical coagulometer (Mitsubishi Chemical Medience, Tokyo, Japan) to measure the prothrombin time (PT) and the activated partial thromboplastin time (aPTT).

### 2.4. Statistical Analysis

We used multivariate logistic regression to compare the differences in the genotype and haplotype frequencies between the RIF patients and controls. Allelic frequencies were assessed for Hardy–Weinberg equilibrium (HWE), using *p* < 0.05 as the significance threshold. We used adjusted odds ratios (AORs) and 95% confidence intervals (CIs) to assess the associations between the different genotypes and RIF; a *p*-value < 0.05 was considered significant. We evaluated the differences in hormone concentrations (E2, FSH, LH, PRL, and TSH), according to *HOTAIR* genotypes and alleles, using a one-way analysis of variance (ANOVA), with a post hoc Scheffé test for all pairwise comparisons, and independent two-sample Student’s *t*-tests, as appropriate. Data are presented as the mean ± standard deviation (SD). Statistical analyses were performed using GraphPad Prism version 4.0 (GraphPad Software, Inc., La Jolla, CA, USA) and StatsDirect version 2.4.4 (StatsDirect Ltd., Altrincham, UK).

## 3. Results

We analyzed 155 RIF patients and 330 healthy controls. Before performing the statistical analysis, we matched the mean age in each group. The mean body mass index was significantly different between controls and patients (*p* < 0.048) and homocysteine levels were also significantly different between groups (Table 1). Additionally, hormonal parameters, including E2 and LH, were significantly different between the controls and RIF patients.

We identified the genotype frequencies of each polymorphism. For rs1899663 and rs7958904, heterozygous genotypes and the dominant model were found to exert protective effects against RIF (heterozygous genotype frequencies: rs1899663, AOR: 0.638, 95% CI: 0.420–0.969, *p* = 0.035; rs7958904, AOR: 0.654, 95% CI: 0.432–0.948, *p* = 0.026). The other SNVs (rs4759314 and rs920778) did not show significant differences between the controls and patients (Table 2). We also analyzed the genotype frequencies among patients according to the numbers of RIFs. The frequencies of rs1899663 and rs7958904 were significantly different between the RIF patients and controls; however, among patients with RIF ≥ 4 expressing the dominant model of rs1899663, no significant difference was found (genotype frequencies for the dominant model of rs1899663 for RIF ≥ 3, AOR: 0.576, 95% CI: 0.387–0.0.917, *p* = 0.017; for RIF ≥ 4: AOR: 0.628, 95% CI: 0.390–1.013, *p* = 0.056, Table 3).

**Figure 1:**
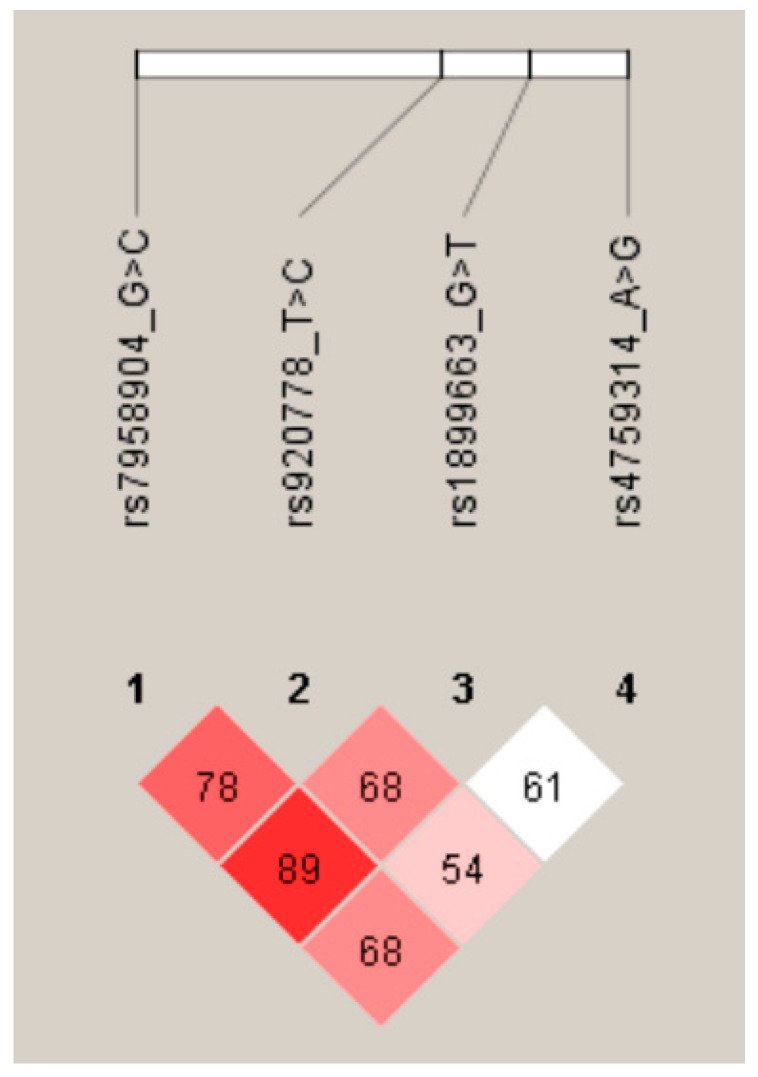
Linkage disequilibrium between *HOTAIR* loci.

In the four-site haplotype analysis (Table 4, Table S2), we identified regularly occurring haplotype patterns. First, all T-C (rs920778T>C/ rs7958904G>C), T-T (rs920778T>C/ rs1899663G>T), A-C (rs4759314A>G/ rs7958904G>C), and A-T (rs4759314A>G/ rs1899663G>T) haplotypes were found to exert protective effects compared with other major allele combinations. Interestingly, the rs1899663G>T/ rs7958904G>C haplotype showed a varying occurrence that appeared to depend on the rs7958904 allele (T-G: OR; 3.170, 95%CI: 1.213–8.284, *p* = 0.013; T-C: OR: 0.610, 95%CI: 0.424–0.879, *p* = 0.008). Similarly, the rs4759314A>G/ rs920778T>C haplotype occurrence appeared to depend on the rs920778 allele (G-T: OR: 0.150, 95% CI: 0.019–1.148, *p* = 0.046; T-C: OR: 0.2.424, 95%CI: 1.135–5.117, *p* = 0.019). We also found a similar result in genotype combination that the rs920778/ rs7958904 (TT/CC) type has protective effects (OR: 0.172, 95% CI: 0.039–0.751, *p* = 0.019) (Table S3). The rs1899663/ rs7958904 (GT/GC) type shows protective effects (OR: 0.571, 95% CI: 0.361–0.903, *p* = 0.017) as determined by the genotype frequency analysis (Table S3). Additionally, in the linkage disequilibrium analysis, we confirmed that rs7958904 and rs1899663 have strong disequilibrium in participants (Figure 1). Among the possible three-allele combinations, the haplotypes A-T-T (rs4759314A>G/ rs920778T>C/ rs1899663G>T), A-T-C (rs4759314A>G/ rs920778T>C/ rs7958904G>C), A-T-C (rs4759314A>G/ rs1899663G>T/ rs7958904G>C), and T-T-C (rs920778T>C/ rs1899663G>T/ rs7958904G>C) were found to be protective, which agrees with the haplotype patterns observed for two-allele combinations. However, rs920778T>C/ rs1899663G>T/ rs7958904G>C was only associated with RIF risk when expressed as the C-T-G haplotype (OR: 4.356, 95% CI: 1.327–14.300, *p* = 0.015). Among the four-allele combinations, the A-T-T-C haplotype (OR: 0.043, 95% CI: 0.005–0.314, *p* < 0.0001) was protective, whereas the A-C-T-G haplotype (OR: 4.345, 95% CI: 1.324–14.260, *p* = 0.015) was associated with RIF risk. Interestingly, we identified the C-G-G haplotype (rs920778T>C/ rs1899663G>T/ rs7958904G>C) as a new protective haplotype against RIF, which was also associated with the protective four-allele combination (A-C-G-G, *p* < 0.05). We also significantly found that the genotype combination *HOTAIR* rs1899663 / *HOTAIR* rs7958904 (GT/GC) type is protective (OR: 0.571, 95% CI: 0.361–0.903, *p* = 0.017).

We performed ANOVA tests to reveal associations between the clinical parameters and genotypes. We found that increased Hgb levels were associated with rs1899663 and rs7958904 polymorphisms among all subjects (Table 5, *p* < 0.05). Although a trend towards increased Hgb levels in RIF patients according to polymorphisms was identified, this relationship was not significant (Table 6). Hormone levels varied according to the identified SNVs. LH levels were significantly different between the rs7958904 genotypes among the total RIF patients (Table 5) and controls (Table 7). A marginal trend toward significance was observed for LH levels depending on the rs920778 and rs7958904 alleles among the RIF patients. (Tables S4–S6).

## 4. Discussion

We tried to identify a correlation between RIF occurrence and the lncRNA *HOTAIR*. Our results showed that the allelic frequencies of the rs1899663 and rs7958904 SNVs were significantly different between the control and RIF patients. These variants have previously been reported in association with other diseases, including various cancers, sclerosis, and psychiatric conditions [23,26,27,28].

*HOTAIR* is a well-known lncRNA. The 5′ end interacts with PRC2, which is associated with histone methyltransferase activity, whereas the 3′ end interacts with LSD1 [29,30]. *HOTAIR* overexpression has been shown to cause gene silencing due to histone modifications. Because of these features, *HOTAIR* has also been associated with cancer development, metastasis, cell cycle, apoptosis, and progression.

Previously, lncRNAs have been associated with placental development [31], suggesting that lncRNA dysfunction could result in various diseases [31]. A great deal of evidence has linked *HOTAIR* with various cancer types, including gastric, colorectal, hepatoma, and esophageal squamous cell carcinoma [13,32,33,34,35,36]. In our previous study, a single nucleotide variation in *HOTAIR* was found to be associated with pathology and mortality in colorectal cancer patients [23], and another study reported an association with cervical cancer and the increased expression of *HOTAIR* in ovarian cancer stem cells [37]. It has been reported that lncRNA *HOTAIR* regulates CCND1 and CCND2 genes [38]. CCND1 gene is the important factor for developing oocytes and meiotic maturation, which is expected to be used in IVF [39].

Overexpression of *HOTAIR* is a risk for the development of estrogen receptor-positive breast cancer. Similarly, overexpression of *HOTAIR* is associated with multi-drug resistance in ovarian cancer patients via inducing NF-κB [22]. Several single nucleotide variants, such as rs920778 and rs12826786, regulate *HOTAIR* expression [40]. A previous report found that *HOTAIR* was highly expressed in several human organ systems [41], including the endometrium, but not in the ovaries. However, these results are dependent on the tissues and cells used [41], and further research is needed to confirm this.

*HOX* gene widely appears in vertebrates as having a role in planning embryonic development [42]. *HOTAIR* is located in the *HOXC* cluster, especially encoded between *HOXC11* and *HOXC12*; as previously reported, *HOTAIR* expression is correlated with *HOXC11* expression, but not *HOXC12* in urothelial cancer cells [43]. Another role of *HOTAIR* is to repress *HOXD* expression, especially *HOXD10* [43]. We suggest that *HOTAIR* expression correlates with RIF occurrence via contributing to *HOXC* and *HOXD* expression in the endometrium.

Results from genomic and functional studies indicate that one of the Polycomb group (PcG), PRC2, is strongly correlated with the presence of CpG islands (CGIs) and causes gene silencing [44]. PRC2 inhibits transcription as well as X-chromosome inactivation (XCI). In mammalian females, XCI is regulated by the XIST gene, which is located in the X-chromosome [45]. XIST is recruited to PRC2 and binds to the X-chromosome, leading to inactivation of the X-chromosome by H3K27me3. XCI is expressed during embryo implantation and is important in proper mammalian development [45,46,47].

The *HOTAIR* promoter regions contain binding sites for estrogen receptor (ER), interferon regulatory factor 1 (IRF1), and NF-κB. Because of the estrogen response region in the *HOTAIR* promoter, overexpression of *HOTAIR* can lead to cell proliferation and growth in the breast cancer cell line MCF-7 [22].

*HOTAIR* has been demonstrated to suppresses placental angiogenesis, proliferation, and invasion [48]. The *HOTAIR* transcript level has also been associated with the occurrence of pre-eclampsia [49]. Many reports have suggested that *HOTAIR* can affect trophoblast invasion, both positively and negatively [49,50]. In various cancers, *HOTAIR* is a well-known promoter of angiogenesis, as well as a promoter of cancer cell proliferation and invasion [12]. Additional investigations remain necessary to confirm the roles played by *HOTAIR*.

Contemporary studies have found that lncRNAs can bind with complementary miRNAs [51], and *HOTAIR* has been shown to contain binding sites that complement specific miRNAs. *HOTAIR* may act as an miRNA sponge, regulating miRNA expression levels. For example, *HOTAIR* can bind to miR-130a, reducing miR-130a levels in gallbladder cancer, and *HOTAIR* expression was also inversely related with miR-124 levels in gastric cancer [11]. *HOTAIR* has also been associated with invasion and metastasis [37]. Moreover, miR-1 has been shown to promote tumorigenicity by upregulating Cyclin D1 (*CCND1*) gene expression, and miR-148a promotes cancer cell invasion and migration through the upregulation of Snail2 [23,33,52].

The absorption of miRNAs in the placenta, endometrium, or ovaries may result in the occurrence of pregnancy-related diseases.

This study has several limitations. Firstly, our data are not generalizable to the wider population because our sample sizes were small. However, we have confirmed the power of our study using a statistical power analysis. Secondly, our study population was limited to Korean individuals; however, the genotypes of each polymorphism examined were confirmed to be in HWE. Additionally, the confirmation of genotypes and allelic frequencies should be confirmed in vitro or tissue such as the placenta or endometrium.

## 5. Conclusions

We analyzed the association between four *HOTAIR* variants and RIF occurrence in a population of Korean women. We discovered two *HOTAIR* SNVs (rs1899663 and rs7958904) that were significantly associated with RIF occurrence. This is the first study to report an association between *HOTAIR* and RIF.

## Figures and Tables

**Table 1:** Clinical profiles of RIF patients and control subjects.

Characteristics	Controls (*n* = 330)	RIF (*n* = 155)	*p*-Value
Age (years)	33.69 ± 2.92	34.07 ± 3.11	0.194
BMI (kg/m²)	21.79 ± 3.40	20.96 ± 2.84	**0.048**
Previous implantation failure (n)	N/A	4.90 ± 2.12	
Live births (n)	1.67 ± 0.57	N/A	
PT (sec)	11.24 ± 3.18	10.78 ± 2.27	0.332
aPTT (sec)	30.26 ± 4.48	29.37 ± 3.48	0.127
PLT (10^3^/µL)	242.52 ± 60.32	237.98 ± 59.63	0.895
Homocysteine (µmol/L)	3.71 ± 4.81	6.79 ± 1.48	**<0.0001**
Folate (mg/mL)	13.67 ± 9.26	15.5 8 ± 10.19	0.617
E2	26.27 ±14.72	37.88 ± 26.09	<0.0001 *
FSH	8.16 ± 2.85	8.88 ± 5.04	0.909 *
LH	3.32 ± 1.76	4.84 ± 2.37	<0.0001 *
Hgb	36.14±4.01	12.56±1.44	<0.0001 *

BMI, body mass index; PT, prothrombin time; aPTT, activated partial thromboplastin time; PLT, platelet; E2, estradiol; FSH, follicle stimulating hormone; LH, luteinizing hormone; N/A, not applicable; RIF, recurrent implantation failure. Previous implantation failure: absence of implantation after ≥ 3 embryo transfers with high-quality embryos. Hgb; hemoglobin. *: Mann-Whitney test.

**Table 2:** Comparison of genotype frequencies and AOR values for polymorphisms between RIF patients and control subjects.

Genotypes	Controls (*n* = 330)	RIF (*n* = 155)	COR (95% CI)	*p*-Value	AOR (95% CI)	*p*-Value
***HOTAIR* rs4759314**						
AA	303 (91.8)	140 (90.3)	1.000 (reference)		1.000 (reference)	
AG	25 (7.6)	15 (9.7)	1.299 (0.664–2.540)	0.445	1.299 (0.663–2.544)	0.445
GG	2 (0.6)	0 (0.0)	N/A	0.996	N/A	0.996
Dominant (AA vs. AG + GG)			1.202 (0.620–2.332	0.585	1.206 (0.621–2.342)	0.580
Recessive (AA + AG vs. GG)			N/A	0.996	N/A	0.996
HWE-P	0.074	0.527				
***HOTAIR* rs920778**						
TT	196 (59.4)	92 (59.4)	1.000 (reference)		1.000 (reference)	
TC	122 (37.0)	55 (35.5)	0.960 (0.641–0.438)	0.845	0.944 (0.629–0.416)	0.781
CC	12 (3.6)	8 (5.2)	1.420 (0.561–0.594)	0.459	1.475 (0.579–3.751)	0.415
Dominant (TT vs. TC + CC)			1.002 (0.679–0.477)	0.994	0.991 (0.671–0.463)	0.964
Recessive (TT + TC vs. CC)			1.442 (0.577–0.603)	0.433	1.525 (0.606–0.833)	0.370
HWE-P	0.185	0.953				
***HOTAIR* rs1899663**						
GG	188 (57.0)	104 (67.1)	1.000 (reference)		1.000 (reference)	
GT	125 (37.9)	45 (29.0)	0.651 (0.429–0.987)	**0.043**	0.638 (0.420–0.969)	**0.035**
TT	17 (5.2)	6 (3.9)	0.638 (0.244–1.668)	0.359	0.625 (0.239–1.639)	0.340
Dominant (GG vs. GT + TT)			0.649 (0.435–0.968)	**0.034**	0.638 (0.427–0.952)	**0.028**
Recessive (GG + GT vs. TT)			0.741 (0.287–1.919)	0.537	0.728 (0.281–1.889)	0.515
HWE-P	0.517	0.684				
***HOTAIR* rs7958904**						
GG	176 (53.3)	99 (63.9)	1.000 (reference)		1.000 (reference)	
GC	129 (39.1)	48 (31.0)	0.662 (0.438–1.000)	**0.050**	0.654 (0.432–0.989)	**0.044**
CC	25 (7.6)	8 (5.2)	0.569 (0.247–1.309)	0.185	0.566 (0.246–1.302)	0.181
Dominant (GG vs. GC + CC)			0.647 (0.437–0.957)	**0.029**	0.640 (0.432–0.948)	**0.026**
Recessive (GG + GC vs. CC)			0.664 (0.292–1.508)	0.328	0.655 (0.288–1.490)	0.313
HWE-P	0.840	0.494				

Note: AOR was adjusted by the age of participants. RIF, recurrent implantation failure; COR, crude odds ratio; AOR, adjusted odds ratio; CI, confidence interval; HWE-P, Hardy–Weinberg equilibrium.

**Table 3:** Genotype frequencies for each polymorphism according to the number of RIFs.

Genotypes	Controls (*n* = 330)	RIF ≥ 3 (*n* = 130)	AOR (95% CI)	*p*-Value	RIF ≥ 4 (*n* = 98)	AOR (95% CI)	*p*-Value
***HOTAIR* rs4759314**							
AA	303 (91.8)	119 (91.5)	1.000 (reference)		88 (89.8)	1.000 (reference)	
AG	25 (7.6)	11 (8.5)	1.123 (0.535–2.359)	0.759	10 (10.2)	1.371 (0.632–2.971)	0.424
GG	2 (0.6)	0 (0.0)	N/A	0.994	0 (0.0)	N/A	0.994
Dominant (GG vs. GA + AA)			1.043 (0.501–2.175)	0.910		1.275 (0.593–2.742)	0.535
Recessive (GG + GA vs. AA)			N/A	0.994		N/A	0.994
***HOTAIR* rs920778**							
TT	196 (59.4)	80 (61.5)	1.000 (reference)		59 (60.2)	1.000 (reference)	
TC	122 (37.0)	43 (33.1)	0.846 (0.547–0.309)	0.452	32 (32.7)	0.854 (0.524–0.391)	0.526
CC	12 (3.6)	7 (5.4)	1.500 (0.565–0.974)	0.415	7 (7.1)	2.086 (0.776–0.604)	0.145
Dominant (CC vs. CT + TT)			0.903 (0.594–0.370)	0.631		0.957 (0.603–0.519)	0.853
Recessive (CC + CT vs. TT)			1.610 (0.614–0.217)	0.332		2.213 (0.837–0.846)	0.109
***HOTAIR* rs1899663**							
GG	188 (57.0)	89 (68.5)	1.000 (reference)		66 (67.3)	1.000 (reference)	
GT	125 (37.9)	35 (26.9)	0.576 (0.365–0.907)	**0.017**	26 (26.5)	0.578 (0.347–0.963)	**0.035**
TT	17 (5.2)	6 (4.6)	0.729 (0.277–1.918)	0.522	6 (6.1)	0.976 (0.368–2.592)	0.962
Dominant (GG vs. GT + TT)			0.596 (0.387–0.917)	**0.019**		0.628 (0.390–1.013)	**0.056**
Recessive (GG + GT vs. TT)			0.873 (0.335-2.272)	0.781		1.174 (0.448–3.076)	0.744
***HOTAIR* rs7958904**							
GG	176 (53.3)	86 (66.2)	1.000 (reference)		65 (66.3)	1.000 (reference)	
GC	129 (39.1)	37 (28.5)	0.582 (0.372–0.912)	**0.018**	26 (26.5)	0.544 (0.327–0.906)	**0.019**
CC	25 (7.6)	7 (5.4)	0.565 (0.235–1.361)	0.203	7 (7.1)	0.745 (0.306–1.809)	0.515
Dominant (GG vs. GC + CC)			0.579 (0.379–0.885)	**0.012**		0.577 (0.360–0.926)	**0.023**
Recessive (GG + GC vs. CC)			0.681 (0.286–1.619)	0.384		0.918 (0.383–2.199)	0.848

Note: AOR was adjusted for the age of participants. RIF, recurrent implantation failure; AOR, adjusted odds ratio; CI, confidence interval.

**Table 4:** Allele combination analysis for the four evaluated polymorphisms in RIF patients and controls subjects.

Allele Combination	Controls (2*n* = 660)	Case (2*n* = 310)	OR (95% CI)	*p*-Value
*HOTAIR* rs4759314A>G/rs920778T>C/rs1899663G>T/rs7958904G>C				
A-T-G-G	0.6811 (450)	0.7512 (233)	1.000 (reference)	
A-T-**T**-**C**	0.0678 (45)	0.0032 (1)	0.043 (0.005–0.314)	**<0.0001**
A-**C**-G-G	0.0293 (19)	0.0033 (1)	0.102 (0.013–0.764)	**0.006**
A-**C**-**T**-G	0.006 (4)	0.0293 (9)	4.345 (1.324–14.260)	**0.015**
*HOTAIR* rs4759314A>G/rs920778T>C/rs1899663G>T				
A-T-G	0.6929 (457)	0.7612 (236)	1.000 (reference)	
A-T-**T**	0.0688 (45)	0.0032 (1)	0.043 (0.005–0.314)	**<0.0001**
A-**C**-**G**	0.0305 (20)	0.0065 (2)	0.194 (0.044–0.836)	**0.012**
*HOTAIR* rs4759314A>G/rs920778T>C/rs7958904G>C				
A-T-G	0.6831 (451)	0.7536 (234)	1.000 (reference)	
A-T-**C**	0.0768 (51)	0.0134 (4)	0.151 (0.053–0.424)	**<0.0001**
*HOTAIR* rs4759314A>G/rs1899663G>T/rs7958904G>C				
A-G-G	0.7105 (469)	0.7576 (235)	1.000 (reference)	
A-**T**-**C**	0.2287 (151)	0.1512 (47)	0.621 (0.432–0.893)	**0.010**
*HOTAIR* rs920778T>C/rs1899663G>T/rs7958904G>C				
T-G-G	0.6868 (453)	0.7543 (234)	1.000 (reference)	
T-**T**-**C**	0.0696 (46)	0.0032 (1)	0.042 (0.005–0.307)	**<0.0001**
**C**-G-G	0.031 (20)	0.0066 (2)	0.194 (0.044–0.836)	**0.012**
**C**-**T**-G	0.0061 (4)	0.0293 (9)	4.356 (1.327–14.300)	**0.015**
*HOTAIR* rs4759314A>G/rs920778T>C				
A-T	0.7573 (500)	0.767 (238)	1.000 (reference)	
**G**-T	0.0215 (14)	0.004 (1)	0.150 (0.019–1.148)	**0.046**
**G**-**C**	0.0194 (13)	0.0476 (15)	2.424 (1.135–5.177)	**0.019**
*HOTAIR* rs4759314A>G/rs1899663G>T				
A-G	0.718 (474)	0.7753 (240)	1.000 (reference)	
A-**T**	0.2381 (157)	0.1763 (55)	0.692 (0.490–0.976)	**0.035**
*HOTAIR* rs4759314A>G/rs7958904G>C				
A-G	0.7192 (475)	0.7859 (244)	1.000 (reference)	
A-**C**	0.2369 (156)	0.1658 (51)	0.636 (0.448–0.905)	**0.011**
*HOTAIR* rs920778T>C/rs1899663G>T				
T-G	0.7017 (463)	0.7642 (237)	1.000 (reference)	
T-**T**	0.077 (51)	0.0068 (2)	0.077 (0.018–0.318)	**<0.0001**
*HOTAIR* rs920778T>C/rs7958904G>C				
T-G	0.691 (456)	0.7576 (235)	1.000 (reference)	
T-**C**	0.0878 (58)	0.0134 (4)	0.134 (0.047–0.373)	**<0.0001**
*HOTAIR* rs1899663G>T/rs7958904G>C				
G-G	0.7177 (474)	0.7594 (235)	1.000 (reference)	
**T**-G	0.0111 (7)	0.0341 (11)	3.170 (1.213–8.284)	**0.013**
**T**-**C**	0.2298 (152)	0.1497 (46)	0.610 (0.424–0.879)	**0.008**

RIF, recurrent implantation failure; OR, odds ratio; CI, confidence interval.

**Table 5:** Differences in the various clinical parameters according to *HOTAIR* gene polymorphisms in RIF patients and control subjects.

Genotypes	Homocysteine (mmol/L)	CD56^+^ NK Cells (%)	PT (sec)	Uric Acid (mg/dl)	T. Chol (mg/dl)	BUN (mg/dl)	Creatinine (mg/dl)	Hgb (mg/dl)	Estradiol (pg/mL)	FSH (mIU/mL)	LH (mIU/mL)
	Mean ± SD (133)	Mean ± SD (132)	Mean ± SD (164)	Mean ± SD (77)	Mean ± SD (126)	Mean ± SD (152)	Mean ± SD (153)	Mean ± SD (277)	Mean ± SD (220)	Mean ± SD (206)	Mean ± SD (200)
***HOTAIR* rs4759314**											
AA	5.17 ± 4.21	18.89 ± 9.56	10.88 ± 2.65	4.01 ± 1.01	190.73 ± 50.34	9.82 ± 2.83	0.76 ± 0.10	27.16 ± 11.99	32.63 ± 22.61	8.37 ± 3.73	4.06 ± 2.16
AG	4.28 ± 2.51	17.39 ± 7.08	11.18 ± 0.54	3.43 ± 0.83	189.18 ± 27.23	11.90 ± 2.39	0.80 ± 0.09	25.97 ± 11.85	26.78 ± 11.33	9.90 ± 6.43	3.78 ± 2.56
GG	2	N/A	8.93 ± 1.59	N/A	N/A	N/A	N/A	34.20 ± 3.95	N/A	N/A	N/A
*P*	0.422	0.572	0.480	0.181	0.920	**0.015**	0.220	0.633	0.308	0.540	0.606
***HOTAIR* rs920778**											
TT	4.61 ± 3.09	19.07 ± 9.98	10.74 ± 2.12	4.07 ± 1.09	188.13 ± 45.24	9.90 ± 2.87	0.77 ± 0.10	26.95 ± 12.08	32.09 ± 20.16	8.60 ± 4.12	4.33 ± 2.22
TC	6.41 ± 5.67	19.15 ± 8.42	11.12 ± 3.21	3.76 ± 0.82	190.61 ± 45.85	10.20 ± 2.91	0.77 ± 0.10	27.52 ± 11.80	32.85 ± 25.28	8.52 ± 4.07	3.58 ± 2.16
CC	3.24 ± 2.47	12.99 ± 5.53	11.07 ± 0.59	3.80 ± 0.80	221.67 ± 96.16	9.43 ± 2.01	0.73 ± 0.08	25.91 ± 11.92	26.95 ± 13.24	6.77 ± 1.77	3.93 ± 1.53
*P*	**0.027**	0.199	0.641	0.434	0.269	0.745	0.752	0.875	0.769	0.462	0.071
***HOTAIR* rs1899663**											
GG	4.62 ± 3.04	19.32 ± 9.74	10.87 ± 1.79	3.98 ± 1.11	186.94 ± 43.30	10.14 ± 2.95	0.77 ± 0.11	25.59 ± 12.21	32.78 ± 19.96	8.56 ± 4.14	4.31 ± 2.29
GT	6.04 ± 5.76	18.54 ± 8.35	10.92 ± 3.66	3.96 ± 0.77	197.25 ± 59.29	9.67 ± 2.74	0.76 ± 0.10	28.93 ± 11.33	32.70 ± 26.08	8.22 ± 3.33	3.75 ± 2.09
TT	4.20 ± 3.11	11.07 ± 4.38	10.80 ± 0.76	3.50 ± 0.71	203.00 ± 33.18	10.08 ± 1.02	0.80 ± 0.08	31.57 ± 10.82	24.20 ± 10.60	9.47 ± 6.20	3.11 ± 1.46
*P*	0.178	0.109	0.991	0.806	0.498	0.636	0.613	**0.033**	0.374	0.551	0.064
***HOTAIR* rs7958904**											
GG	4.80 ± 3.05	19.47 ± 9.87	10.85 ± 1.85	4.00 ± 1.07	191.16 ± 49.92	9.90 ± 2.91	0.77 ± 0.11	25.35 ± 12.28	33.87 ± 23.50	8.75 ± 4.57	4.46 ± 2.38
GC	5.65 ± 5.96	18.35 ± 8.34	11.13 ± 3.26	3.83 ± 0.87	190.22 ± 48.77	10.17 ± 2.86	0.76 ± 0.11	29.14 ± 11.27	31.65 ± 21.19	8.31 ± 3.48	3.43 ± 1.86
CC	4.40 ± 2.73	12.88 ± 6.00	9.26 ± 4.09	4.20 ± 0.57	184.00 ±30.31	10.08 ± 1.73	0.78 ± 0.04	30.82 ± 10.46	23.63 ± 11.63	7.76 ± 2.23	3.75 ± 1.72
*P*	0.504	0.153	0.215	0.756	0.950	0.866	0.733	**0.016**	0.164	0.548	0.007

Note: RIF, recurrent implantation failure; NK, natural killer; PLT, platelet count; PT, prothrombin time; T.chol, total cholesterol; BUN, blood urea nitrogen; Hgb, hemoglobin; FSH, follicle-stimulating hormone; LH, luteinizing hormone; SD, standard deviation; N/A, not applicable.

**Table 6:** Differences in clinical parameters according to *HOTAIR* gene polymorphisms among RIF patients.

Genotypes	Homocysteine (mmol/L)	PLT (10^3^/µL)	aPTT (sec)	PT (sec)	Uric Acid (mg/dl)	BUN (mg/dl)	Creatinine (mg/dl)	Hgb (mg/dl)	Estradiol (pg/mL)	FSH (mIU/mL)	LH (mIU/mL)
	Mean ± SD (57)	Mean ± SD (128)	Mean ± SD (127)	Mean ± SD (127)	Mean ± SD (70)	Mean ± SD (122)	Mean ± SD (123)	Mean ± SD (106)	Mean ± SD(111)	Mean ± SD (97)	Mean ± SD (94)
***HOTAIR* rs4759314**											
AA	6.82 ± 1.51	240.15 ± 60.63	29.30 ± 3.33	10.73 ± 2.38	4.05 ± 1.00	10.28 ± 2.86	0.78 ± 0.10	12.48 ± 1.47	39.28 ± 27.04	8.65 ± 4.52	4.91 ± 2.32
AG	6.54 ± 1.33	215.00 ± 43.31	29.95 ± 4.76	11.27 ± 0.57	3.43 ± 0.83	12.26 ± 2.13	0.81 ± 0.08	13.31 ± 0.72	27.44 ± 12.13	10.71 ± 8.13	4.32 ± 2.75
GG	N/A	N/A	N/A	N/A	N/A	N/A	N/A	N/A	N/A	N/A	N/A
*P*	0.666	0.182	0.525	0.413	0.145	**0.027**	0.403	0.082	0.198	0.955	0.443
***HOTAIR* rs920778**											
TT	6.53 ± 1.31	242.20 ± 63.76	29.58 ± 3.58	10.72 ± 2.37	4.14 ± 1.08	10.42 ± 2.93	0.79 ± 0.10	12.43 ± 1.48	37.83 ± 23.07	8.92 ± 5.04	4.92 ± 2.35
TC	7.17 ± 1.70	233.86 ± 53.68	29.14 ± 3.30	10.85 ± 2.23	3.77 ± 0.79	10.58 ± 2.86	0.78 ± 0.10	12.71 ± 1.35	40.86 ± 32.45	9.34 ± 5.41	4.81 ± 2.56
CC	8.12	207.60 ± 30.57	28.22 ± 4.04	11.22 ± 0.49	3.40 ± 0.57	9.88 ± 1.89	0.76 ± 0.05	13.00 ± 1.65	27.48 ± 15.55	6.06 ± 0.97	4.28 ± 1.62
*P*	0.193	0.389	0.603	0.867	0.244	0.863	0.826	0.508	0.506	0.343	0.818
***HOTAIR* rs1899663**											
GG	6.52 ± 1.29	240.84 ± 63.68	29.73 ± 3.77	10.90 ± 1.97	4.34 ± 1.09	10.64 ± 2.95	0.79 ± 0.10	12.54 ± 1.45	37.39 ± 22.13	8.77 ± 4.87	4.88 ± 2.34
GT	7.44 ± 1.74	233.97 ± 50.45	28.69 ± 2.78	10.51 ± 2.90	3.96 ± 0.72	10.12 ± 2.74	0.78 ± 0.10	12.57 ± 1.38	41.05 ± 35.07	8.72 ± 4.11	4.99 ± 2.50
TT	N/A	206.00 ± 46.13	27.70 ± 0.66	10.80 ± 0.76	3.50 ± 0.71	9.80 ± 1.06	0.80 ± 0.10	12.90 ± 2.26	31.58 ± 6.98	11.73 ± 11.77	3.34 ± 2.02
*P*	**0.031**	0.543	0.222	0.678	0.743	0.601	0.871	0.914	0.632	0.842	0.426
***HOTAIR* rs7958904**											
GG	6.54 ± 1.28	245.49 ± 62.17	29.62 ± 3.59	10.89 ± 2.00	4.03 ± 1.06	10.42 ± 2.94	0.79 ± 0.10	12.48 ± 1.45	38.93 ± 26.86	9.14 ± 5.45	4.97 ± 2.51
GC	7.52 ± 1.87	233.42 ± 55.35	28.91 ± 3.18	10.77 ± 2.42	3.89 ± 0.84	10.62 ± 2.83	0.78 ± 0.10	12.57 ± 1.45	39.20 ± 26.71	8.83 ± 4.42	4.63 ± 2.15
CC	7.10 ± 1.57	217.83 ± 12.89	28.64 ± 4.15	9.10 ± 4.55	4.20 ± 0.57	9.92 ± 1.88	0.78 ± 0.04	13.66 ± 0.67	26.08 ± 14.87	6.27 ± 1.18	4.36 ± 1.68
*P*	0.099	0.122	0.526	0.232	0.847	0.858	0.737	0.206	0.508	0.413	0.743

Note: RIF, recurrent implantation failure; PLT, platelet count; aPTT, activated partial thromboplastin time; PT, prothrombin time; BUN, blood urea nitrogen; Hgb, hemoglobin; FSH, follicle-stimulating hormone; LH, luteinizing hormone; N/A, not applicable.

**Table 7:** Differences in various clinical parameters according to *HOTAIR* gene polymorphisms in control subjects.

Genotypes	Homocysteine (mmol/L)	PLT (10^3^/µL)	aPTT (sec)	PT (sec)	T. Chol (mg/dl)	BUN (mg/dl)	Creatinine (mg/dl)	Hgb (mg/dl)	Estradiol (pg/mL)	FSH (mIU/mL)	LH (mIU/mL)
	Mean ± SD (76)	Mean ± SD (175)	Mean ± SD (67)	Mean ± SD (37)	Mean ± SD (9)	Mean ± SD (30)	Mean ± SD (30)	Mean ± SD (171)	Mean ± SD (109)	Mean ± SD (109)	Mean ± SD (106)
***HOTAIR* rs4759314**											
AA	3.90 ± 5.11	243.31 ± 59.20	30.05 ± 4.49	11.44 ± 3.47	229.44 ± 90.57	8.07 ± 1.86	0.68 ± 0.08	36.20 ± 3.98	26.30 ± 14.97	8.13 ± 2.91	3.35 ± 1.74
AG	2.59 ± 1.66	234.39 ± 78.94	32.32 ± 4.98	10.93 ± 0.38	N/A	7.90	0.7	35.72 ± 4.56	25.93 ± 11.12	8.64 ± 1.97	2.91 ± 2.14
GG	2	232.50 ±7.78	30.35 ± 1.63	8.93 ± 1.59	N/A	N/A	N/A	34.20 ± 3.96	N/A	N/A	N/A
*P*	0.680	0.854	0.504	0.554	N/A	0.931	0.806	0.726	0.949	0.648	0.530
***HOTAIR* rs920778**											
TT	3.29 ± 3.28	239.28 ± 54.86	30.18 ± 4.63	10.81 ± 0.96	183.67 ± 42.30	8.09 ± 1.71	0.69 ± 0.08	36.16 ± 4.19	26.07 ± 14.48	8.30 ± 3.00	3.76 ± 1.94
TC	5.47 ± 8.30	248.08 ± 67.46	30.58 ± 4.40	12.40 ± 6.00	276.50 ± 84.15	8.09 ± 2.33	0.66 ± 0.07	36.22 ± 3.69	26.58 ± 15.49	7.95 ± 2.71	2.77 ± 1.35
CC	2.63 ± 1.77	239.86 ± 74.61	29.78 ± 4.05	10.82 ± 0.77	410.00	7.2	0.60	35.13 ± 4.57	25.35 ± 3.32	8.90 ± 2.26	2.90 ± 0.71
*P*	0.216	0.654	0.928	0.415	0.015	0.899	0.438	0.791	0.981	0.772	0.015
***HOTAIR* rs1899663**											
GG	3.16± 3.20	237.89 ± 56.67	30.18 ± 4.59	10.76 ± 0.85	174.60 ± 40.25	7.88 ± 1.55	0.68 ± 0.08	36.16 ± 4.15	26.57 ± 14.63	8.29 ± 2.98	3.58 ± 2.03
GT	4.97 ± 7.41	248.58 ± 66.51	30.52 ± 4.43	12.13 ± 5.26	298.00 ± 91.85	8.10 ± 2.19	0.67 ± 0.08	35.97 ± 4.06	27.01 ± 15.59	7.94 ± 2.81	3.11 ± 1.50
TT	4.20 ± 3.11	240.90 ± 44.42	27.70	N/A	N/A	10.9	0.80	37.17 ± 1.97	21.25 ± 10.59	8.57 ± 2.48	3.02 ± 1.30
*P*	0.336	0.528	0.815	0.215	**0.029**	0.283	0.324	0.677	0.525	0.741	0.364
***HOTAIR* rs7958904**											
GG	3.36 ± 3.35	241.63 ± 55.35	29.56 ± 4.39	10.70 ± 0.94	216.00 ± 93.86	7.79 ± 1.52	0.68 ± 0.08	36.26 ± 3.94	26.43 ± 14.78	8.22 ± 3.03	3.79 ± 2.02
GC	4.57 ± 7.25	244.65 ± 68.76	31.43 ± 4.61	11.98 ± 4.70	276.50 ± 84.15	8.36 ± 2.32	0.68 ± 0.08	35.90 ± 4.32	27.15 ± 15.75	8.03 ± 2.85	2.80 ± 1.33
CC	3.10 ± 2.20	237.60 ± 46.48	29.60 ± 2.69	10.05	N/A	10.9	0.80	36.54 ± 2.84	22.50 ± 10.30	8.45 ± 2.28	3.47 ± 1.73
*P*	0.594	0.902	0.262	0.466	0.442	0.218	0.327	0.789	0.602	0.878	0.022

Note: RIF, recurrent implantation failure; PLT, platelet count; aPTT, activated partial thromboplastin time; PT, prothrombin time; BUN, blood urea nitrogen; Hgb, hemoglobin; FSH, follicle-stimulating hormone; LH, luteinizing hormone; N/A, not applicable.

## Data Availability

No new data were created or analyzed in this study. Data sharing is not applicable to this article.

## Supplementary Materials

The following are available online at https://www.mdpi.com/1422-0067/22/6/3021/s1, Table S1: Details of miRNA polymorphisms for PCR-RFLP analysis, Table S2: Allele combination analysis of four gene polymorphisms in RIF and controls subjects, Table S3: Genotype combination analysis for the HOTAIR polymorphisms in patients and controls, Table S4: Differences of various clinical parameters according to HOTAIR gene polymorphisms in RIF patient and control subjects. Table S5: Differences of various clinical parameters according to HOTAIR gene polymorphisms in RIF women. Table S6: Differences of various clinical parameters according to HOTAIR gene polymorphisms in control
