# Supplementary material for: Association between HOX Transcript Antisense RNA Single-Nucleotide Variants and Recurrent Implantation Failure
Source: Int J Mol Sci. 2021 Mar 16;22(6):3021. doi: 10.3390/ijms22063021 (PMC8002254; doi:10.3390/ijms22063021)
Supplement: Supplementary file 1 [file ijms-22-03021-s001.pdf]

Supplementary materials

Supplementary Table 1. Details of miRNA polymorphisms for PCR-RFLP analysis

rs number		primer	restriction enzyme
rs1899663	forward	5'- TTT TCC AGT TGA GGA GGG TGG A -3'	*Hph*I
	reverse	5'- CTA ATG GCA AGG GAA GGG AAG G -3'	
rs4759314	forward	5'- ACC CAA AAC CAT TTC CTG AGA G -3'	*Alu*I
	reverse	5'- TTC AGG TTT TAT TAA CTT GCA TCA GC -3'	

Supplemantary Table 2. Allele combination analysis of four gene polymorphisms in RIF and controls subjects.

Haplotype	Controls (2n=660)	Case (2n=310)	OR (95% CI)	*P*-value
HOTAIR rs4759314A>G/rs920778 T>C/rs1899663 G>T/rs7958904 G>C				
A-T-G-G	0.681	0.751	1.000 (reference)	
A-T-G-C	0.008	0.010	1.159 (0.274 - 4.893)	1.000
A-T-T-G	0.005	0.000	0.276 (0.014 - 5.362)	0.555
A-T-T-C	0.068	0.003	0.043 (0.005 - 0.314)	**<0.0001**
A-C-G-G	0.029	0.003	0.102 (0.013 - 0.764)	**0.006**
A-C-G-C	0.001	0.003	1.931 (0.120 - 31.040)	1.000
A-C-T-G	0.006	0.029	4.345 (1.324 - 14.260)	**0.015**
A-C-T-C	0.161	0.148	0.838 (0.572 - 1.226)	0.363
G-T-G-G	0.006	0.003	0.483 (0.053 - 4.347)	0.666
G-T-G-C	0.009	0.000	0.148 (0.008 - 2.648)	0.102
G-T-T-G	0.000	0.003	5.788 (0.234 - 142.700)	0.342
G-T-T-C	0.002	0.000	0.643 (0.026 - 15.860)	1.000
G-C-G-G	0.002	0.003	1.931 (0.120 - 31.040)	1.000
G-C-G-C	0.022	0.042	1.674 (0.783 - 3.577)	0.179
G-C-T-G	0.000	0.000	N/A	N/A
G-C-T-C	0.000	0.000	N/A	N/A
HOTAIR rs4759314A>G/rs920778 T>C/rs1899663 G>T				
A-T-G	0.6929	0.7612	1.000 (reference)	
A-T-T	0.0688	0.0032	0.043 (0.005 - 0.314)	**<0.0001**
A-C-G	0.0305	0.0065	0.194 (0.044 - 0.836)	**0.012**
A-C-T	0.1669	0.1774	0.968 (0.675 - 1.388)	0.860
G-T-G	0.0119	0.0032	0.242 (0.030 - 1.948)	0.286
G-T-T	0.0052	0.0032	0.646 (0.066 - 6.243)	1.000
G-C-G	0.0238	0.0451	1.694 (0.812 - 3.532)	**0.155**
HOTAIR rs4759314A>G/rs920778 T>C/rs7958904 G>C				
A-T-G	0.683	0.754	1.000 (reference)	
A-T-C	0.077	0.013	0.151 (0.053 - 0.424)	**<0.0001**
A-C-G	0.036	0.030	0.723 (0.330 - 1.580)	0.414
A-C-C	0.163	0.152	0.839 (0.575 - 1.223)	0.360
G-T-G	0.008	0.004	0.386 (0.044 - 3.320)	0.670
G-T-C	0.011	0.000	0.128 (0.007 - 2.259)	0.102
G-C-G	0.002	0.006	3.855 (0.347 - 42.760)	0.272
G-C-C	0.020	0.041	1.927 (0.879 - 4.226)	0.096
HOTAIR rs4759314A>G/rs1899663 G>T/rs7958904 G>C				
A-G-G	0.711	0.758	1.000 (reference)	
A-G-C	0.006	0.013	1.996 (0.495 - 8.053)	0.452
A-T-G	0.011	0.029	2.566 (0.944 - 6.977)	0.056
A-T-C	0.229	0.151	0.621 (0.432 - 0.893)	**0.010**
G-G-G	0.008	0.003	0.399 (0.046 - 3.438)	0.669
G-G-C	0.035	0.042	1.128 (0.561 - 2.267)	0.735
G-T-G	0.000	0.003	5.981 (0.243 - 147.500)	0.335
G-T-C	0.002	0.000	0.665 (0.027 - 16.390)	1.000
HOTAIR rs920778 T>C/rs1899663 G>T/rs7958904 G>C				
T-G-G	0.6868	0.7543	1.000 (reference)	
T-G-C	0.0176	0.0100	0.484 (0.135 - 1.732)	0.408
T-T-G	0.0049	0.0034	0.645 (0.066 - 6.241)	1.000

T-T-C	0.0696	0.0032	0.042 (0.005 - 0.307)	**<0.0001**
C-G-G	0.0310	0.0066	0.194 (0.044 - 0.836)	**0.012**
C-G-C	0.0238	0.0452	1.694 (0.812 - 3.531)	0.155
C-T-G	0.0061	0.0293	4.356 (1.327 - 14.300)	**0.015**
C-T-C	0.160	0.148	0.840 (0.574 - 1.229)	0.369
HOTAIR rs4759314A>G/rs920778 T>C				
A-T	0.757	0.767	1.000 (reference)	
A-C	0.202	0.181	0.885 (0.624 - 1.253)	0.490
G-T	0.022	0.004	0.150 (0.019 - 1.148)	0.046
G-C	0.019	0.048	2.424 (1.135 - 5.177)	**0.019**
HOTAIR rs4759314A>G/rs1899663 G>T				
A-G	0.718	0.775	1.000 (reference)	
A-T	0.238	0.176	0.692 (0.490 - 0.976)	**0.035**
G-G	0.041	0.041	0.951 (0.482 - 1.877)	0.885
G-T	0.003	0.008	1.975 (0.276 - 14.110)	0.606
HOTAIR rs4759314A>G/rs7958904 G>C				
A-G	0.719	0.786	1.000 (reference)	
A-C	0.237	0.166	0.636 (0.448 - 0.905)	**0.011**
G-G	0.010	0.008	0.649 (0.130 - 3.240)	0.723
G-C	0.034	0.041	1.100 (0.548 - 2.210)	0.788
HOTAIR rs920778 T>C/rs1899663 G>T				
T-G	0.702	0.764	1.000 (reference)	
T-T	0.077	0.007	0.077 (0.018 - 0.318)	**<0.0001**
C-G	0.057	0.052	0.823 (0.449 - 1.506)	0.526
C-T	0.164	0.177	0.995 (0.693 - 1.427)	0.978
HOTAIR rs920778 T>C/rs7958904 G>C				
T-G	0.691	0.758	1.000 (reference)	
T-C	0.088	0.013	0.134 (0.047 - 0.373)	**<0.0001**
C-G	0.038	0.036	0.854 (0.412 - 1.766)	0.669
C-C	0.183	0.193	0.962 (0.679 - 1.362)	0.828
HOTAIR rs1899663 G>T/rs7958904 G>C				
G-G	0.718	0.759	1.000 (reference)	
G-C	0.041	0.057	1.345 (0.726 - 2.491)	0.345
T-G	0.011	0.034	3.170 (1.213 - 8.284)	**0.013**
T-C	0.230	0.150	0.610 (0.424 - 0.879)	**0.008**

AOR = odds ratio; *CI* = confidence interval.

a Fisher's exact test; b FDR-adjusted P value.

Supplemantary Table 3. Genotype combination analysis for the HOTAIR polymorphisms in patients and controls

Genotype combination	Controls (n=330)	RIF patients (n=155)	AOR (95% CI)	*P*-value
HOTAIR rs4759314 / HOTAIR rs920778				
AA/TT	187 (56.7)	92 (59.4)	1.000 (reference)	
AA/TC	108 (32.7)	43 (27.7)	0.799 (0.516 - 0.236)	0.313
AA/CC	10 (3.0)	5 (3.2)	1.068 (0.353 - 0.232)	0.907
AG/TT	7 (2.1)	1 (0.6)	0.268 (0.032 - 0.239)	0.224
AG/TC	14 (4.2)	11 (7.1)	1.614 (0.703 - 0.701)	0.259
AG/CC	2 (0.6)	3 (1.9)	3.199 (0.521 - 0.612)	0.209
GG/TT	2 (0.6)	0 (0.0)	N/A	N/A
GG/TC	0 (0.0)	0 (0.0)	N/A	N/A
GG/CC	0 (0.0)	0 (0.0)	N/A	N/A
HOTAIR rs4759314 / HOTAIR rs1899663				
AA/GG	167 (50.6)	93 (60.0)	1.000 (reference)	
AA/GT	120 (36.4)	42 (27.1)	0.616 (0.399 - 0.952)	**0.029**
AA/TT	16 (4.8)	5 (3.2)	0.551 (0.195 - 1.556)	0.261
AG/GG	19 (5.8)	11 (7.1)	1.037 (0.472 - 2.277)	0.928
AG/GT	5 (1.5)	3 (1.9)	1.060 (0.247 - 4.548)	0.937
AG/TT	1 (0.3)	1 (0.6)	1.810 (0.112 - 29.300)	0.676
GG/GG	2 (0.6)	0 (0.0)	N/A	0.995
GG/GT	0 (0.0)	0 (0.0)	N/A	N/A
GG/TT	0 (0.0)	0 (0.0)	N/A	N/A
HOTAIR rs4759314 / HOTAIR rs7958904				
AA/GG	172 (52.1)	97 (62.6)	1.000 (reference)	
AA/GC	113 (34.2)	38 (24.5)	0.588 (0.377 - 0.918)	**0.020**
AA/CC	18 (5.5)	5 (3.2)	0.489 (0.176 - 1.360)	0.171
AG/GG	3 (0.9)	2 (1.3)	1.173 (0.192 - 7.162)	0.863
AG/GC	16 (4.8)	10 (6.5)	1.113 (0.486 - 2.551)	0.800
AG/CC	6 (1.8)	3 (1.9)	0.877 (0.214 - 3.591)	0.855
GG/GG	1 (0.3)	0 (0.0)	N/A	0.995
GG/GC	0 (0.0)	0 (0.0)	N/A	N/A
GG/CC	1 (0.3)	0 (0.0)	N/A	0.995
HOTAIR rs920778 / HOTAIR rs1899663				
TT/GG	165 (50.0)	91 (58.7)	1.000 (reference)	
TT/GT	17 (5.2)	1 (0.6)	0.109 (0.014 - 0.830)	**0.032**
TT/TT	14 (4.2)	0 (0.0)	N/A	N/A
TC/GG	16 (4.8)	13 (8.4)	1.485 (0.683 - 0.229)	0.318
TC/GT	104 (31.5)	41 (26.5)	0.705 (0.452 - 0.100)	0.123
TC/TT	2 (0.6)	1 (0.6)	0.889 (0.079 - 0.975)	0.924
CC/GG	7 (2.1)	0 (0.0)	N/A	N/A
CC/GT	4 (1.2)	3 (1.9)	1.386 (0.302 - 0.347)	0.675
CC/TT	1 (0.3)	5 (3.2)	9.045 (1.040 - 0.654)	**0.046**

HOTAIR rs920778 / HOTAIR rs7958904				
TT/GG	164 (49.7)	90 (58.1)	1.000 (reference)	
TT/GC	11 (3.3)	0 (0.0)	N/A	N/A
TT/CC	21 (6.4)	2 (1.3)	0.172 (0.039 - 0.751)	**0.019**
TC/GG	4 (1.2)	9 (5.8)	4.065 (1.215 - 0.588)	**0.023**
TC/GC	116 (35.2)	46 (29.7)	0.715 (0.465 - 0.097)	0.125
TC/CC	2 (0.6)	0 (0.0)	N/A	N/A
CC/GG	8 (2.4)	0 (0.0)	N/A	N/A
CC/GC	2 (0.6)	2 (1.3)	1.814 (0.251 - 0.110)	0.555
CC/CC	2 (0.6)	6 (3.9)	5.485 (1.083 - 0.773)	**0.040**
HOTAIR rs1899663 / HOTAIR rs7958904				
GG/GG	169 (51.2)	92 (59.4)	1.000 (reference)	
GG/GC	18 (5.5)	11 (7.1)	1.127 (0.510 - 2.491)	0.768
GG/CC	1 (0.3)	1 (0.6)	1.819 (0.112 - 29.46)	0.674
GT/GG	7 (2.1)	6 (3.9)	1.524 (0.495 - 4.692)	0.463
GT/GC	111 (33.6)	35 (22.6)	0.571 (0.361 - 0.903)	**0.017**
GT/CC	7 (2.1)	4 (2.6)	1.039 (0.296 - 3.650)	0.952
TT/GG	0 (0.0)	1 (0.6)	N/A	N/A
TT/GC	0 (0.0)	2 (1.3)	N/A	N/A
TT/CC	17 (5.2)	3 (1.9)	0.321 (0.092 - 1.125)	0.076
HOTAIR rs920778 / HOTAIR rs1899663				
TT/GG	165 (50.0)	91 (58.7)	1.000 (reference)	
TT/GT	17 (5.2)	1 (0.6)	0.109 (0.014 - 0.830)	**0.032**
TT/TT	14 (4.2)	0 (0.0)	N/A	N/A
TC/GG	16 (4.8)	13 (8.4)	1.485 (0.683 - 0.229)	0.318
TC/GT	104 (31.5)	41 (26.5)	0.705 (0.452 - 0.100)	0.123
TC/TT	2 (0.6)	1 (0.6)	0.889 (0.079 - 0.975)	0.924
CC/GG	7 (2.1)	0 (0.0)	N/A	N/A
CC/GT	4 (1.2)	3 (1.9)	1.386 (0.302 - 0.347)	0.675
CC/TT	1 (0.3)	5 (3.2)	9.045 (1.040 - 0.654)	**0.046**
HOTAIR rs920778 / HOTAIR rs7958904				
TT/GG	164 (49.7)	90 (58.1)	1.000 (reference)	
TT/GC	11 (3.3)	0 (0.0)	N/A	N/A
TT/CC	21 (6.4)	2 (1.3)	0.172 (0.039 - 0.751)	**0.019**
TC/GG	4 (1.2)	9 (5.8)	4.065 (1.215 - 0.588)	**0.023**
TC/GC	116 (35.2)	46 (29.7)	0.715 (0.465 - 0.097)	0.125
TC/CC	2 (0.6)	0 (0.0)	N/A	N/A
CC/GG	8 (2.4)	0 (0.0)	N/A	N/A
CC/GC	2 (0.6)	2 (1.3)	1.814 (0.251 - 0.110)	0.555
CC/CC	2 (0.6)	6 (3.9)	5.485 (1.083 - 0.773)	**0.040**
HOTAIR rs1899663 / HOTAIR rs7958904				
GG/GG	169 (51.2)	92 (59.4)	1.000 (reference)	
GG/GC	18 (5.5)	11 (7.1)	1.127 (0.510 - 2.491)	0.768
GG/CC	1 (0.3)	1 (0.6)	1.819 (0.112 - 29.46)	0.674
GT/GG	7 (2.1)	6 (3.9)	1.524 (0.495 - 4.692)	0.463

GT/GC	111 (33.6)	35 (22.6)	0.571 (0.361 - 0.903)	**0.017**
GT/CC	7 (2.1)	4 (2.6)	1.039 (0.296 - 3.650)	0.952
TT/GG	0 (0.0)	1 (0.6)	N/A	N/A
TT/GC	0 (0.0)	2 (1.3)	N/A	N/A
TT/CC	17 (5.2)	3 (1.9)	0.321 (0.092 - 1.125)	0.076

AOR = odds ratio; *CI* = confidence interval. a Fisher's exact test; b FDR-adjusted P value.

Supplementary Table 4. Differences of various clinical parameters according to HOTAIR gene polymorphisms in RIF patient and control subjects.

Genotypes	BMI (kg/m^2)	Homocysteine (mmol/L)	Folate (mg/ml)	PLT (10^3/μℓ)	aPTT (sec)	CD56+ NK cells (%)	PT (sec)	Uric Acid (mg/dl)	T.chol (mg/dl)	BUN (mg/dl)	Creatinine (mg/dl)	Hgb (mg/dl)	Estradiol (pg/ml)	FSH (mIU/ml)	LH (mIU/ml)
	Mean ± SD (230)	Mean ± SD (133)	Mean ± SD (65)	Mean ± SD (303)	Mean ± SD (194)	Mean ± SD (132)	Mean ± SD (164)	Mean ± SD (77)	Mean ± SD (126)	Mean ± SD (152)	Mean ± SD (153)	Mean ± SD (277)	Mean ± SD (220)	Mean ± SD (206)	Mean ± SD (200)
**HOTAIR rs4759314**															
AA	21.26±3.16	5.17±4.21	14.40±8.34	241.97±59.72	29.56±3.77	18.89±9.56	10.88±2.65	4.01±1.01	190.73±50.34	9.82±2.83	0.76±0.10	27.16±11.99	32.63±22.61	8.37±3.73	4.06±2.16
AG	21.16±2.12	4.28±2.51	23.19±18.26	225.50±64.53	30.70±4.82	17.39±7.08	11.18±0.54	3.43±0.83	189.18±27.23	11.90±2.39	0.80±0.09	25.97±11.85	26.78±11.33	9.90±6.43	3.78±2.56
GG	22.94±4.76	2.00±0.00	N/A	232.50±7.78	30.35±1.63	N/A	8.93±1.59	N/A	N/A	N/A	N/A	34.20±3.95	N/A	N/A	N/A
P^a	0.739	0.422	0.353	0.428	0.461	0.572	0.480	0.181	0.920	0.015	0.220	0.633	0.308	0.540	0.606
**HOTAIR rs920778**															
TT	21.25±3.05	4.61±3.09	15.08±8.41	240.54±58.72	29.80±4.00	19.07±9.98	10.74±2.12	4.07±1.09	188.13±45.24	9.90±2.87	0.77±0.10	26.95±12.08	32.09±20.16	8.60±4.12	4.33±2.22
TC	21.26±2.96	6.41±5.67	15.57±12.55	242.29±62.34	29.55±3.66	19.15±8.42	11.12±3.21	3.76±0.82	190.61±45.85	10.20±2.91	0.77±0.10	27.52±11.80	32.85±25.28	8.52±4.07	3.58±2.16
CC	21.53±4.14	3.24±2.47	18.24±14.21	226.42±60.43	28.91±3.87	12.99±5.53	11.07±0.59	3.80±0.80	221.67±96.16	9.43±2.01	0.73±0.08	25.91±11.92	26.95±13.24	6.77±1.77	3.93±1.53
P^a	0.948	0.027	0.905	0.686	0.760	0.199	0.641	0.434	0.269	0.745	0.752	0.875	0.769	0.462	0.071
**HOTAIR rs1899663**															
GG	21.22±3.02	4.62±3.04	15.51±10.44	239.32±60.03	29.87±4.04	19.32±9.74	10.87±1.79	3.98±1.11	186.94±43.30	10.14±2.95	0.77±0.11	25.59±12.21	32.78±19.96	8.56±4.14	4.31±2.29
GT	21.31±3.02	6.04±5.76	14.01±8.36	243.58±61.65	29.40±3.59	18.54±8.35	10.92±3.66	3.96±0.77	197.25±59.29	9.67±2.74	0.76±0.10	28.93±11.33	32.70±26.08	8.22±3.33	3.75±2.09
TT	21.94±4.95	4.20±3.11	35.05	232.85±45.48	27.70±0.54	11.07±4.38	10.80±0.76	3.50±0.71	203.00±33.18	10.08±1.02	0.80±0.08	31.57±10.82	24.20±10.60	9.47±6.20	3.11±1.46
P^a	0.806	0.178	0.121	0.752	0.431	0.109	0.991	0.806	0.498	0.636	0.613	0.033	0.374	0.551	0.064
**HOTAIR rs7958904**															
GG	21.28±3.07	4.80±3.05	24.80±12.36	243.53±58.68	29.60±3.5	19.47±9.87	10.85±1.85	4.00±1.07	191.16±49.92	9.90±2.91	0.77±0.11	25.35±12.28	33.87±23.50	8.75±4.57	4.46±2.38
GC	21.09±2.95	5.65±5.96	11.49±5.28	237.57±65.12	29.91±3.98	18.35±8.34	11.13±3.26	3.83±0.87	190.22±48.77	10.17±2.86	0.76±0.11	29.14±11.27	31.65±21.19	8.31±3.48	3.43±1.86
CC	22.17±4.06	4.40±2.73	16.07±10.74	231.95±40.46	28.91±3.60	12.88±6.00	9.26±4.09	4.20±0.57	184.00±30.31	10.08±1.73	0.78±0.04	30.82±10.46	23.63±11.63	7.76±2.23	3.75±1.72
P^a	0.534	0.504	0.070	0.570	0.761	0.153	0.215	0.756	0.950	0.866	0.733	0.016	0.164	0.548	0.007

Note:BMI,body mass index; PLT, platelet count; aPTT, Activated Partial Thromboplastin Time; PT, prothrombin time; T.chol, Total cholesterol; BUN, blood urea nitrogen; Hgb, hemoglobin

Supplementary Table 5. Differences of various clinical parameters according to HOTAIR gene polymorphisms in RIF women.

Genotypes	BMI (kg/m²)	Homocysteine (mmol/L)	Folate (mg/ml)	PLT ($10^3/\mu\ell$)	aPTT (sec)	CD56+ NK cells (%)	PT (sec)	Uric Acid (mg/dl)	T.chol (mg/dl)	BUN (mg/dl)	Creatinine (mg/dl)	Hgb (mg/dl)	Estradiol (pg/ml)	FSH (mIU/ml)	LH (mIU/ml)
	Mean ± SD (144)	Mean ± SD (57)	Mean ± SD (57)	Mean ± SD (128)	Mean ± SD (127)	Mean ± SD (132)	Mean ± SD (127)	Mean ± SD (70)	Mean ± SD (117)	Mean ± SD (122)	Mean ± SD (123)	Mean ± SD (106)	Mean ± SD(111)	Mean ± SD (97)	Mean ± SD (94)
**HOTAIR rs4759314**															
AA	20.91±2.92	6.82±1.51	14.52±8.28	240.15±60.63	29.30±3.33	18.89±9.56	10.73±2.38	4.05±1.00	187.44±44.58	10.28±2.86	0.78±0.10	12.48±1.47	39.28±27.04	8.65±4.52	4.91±2.32
AG	21.41±1.99	6.54±1.33	23.19±18.26	215.00±43.31	29.95±4.76	17.39±7.08	11.27±0.57	3.43±0.83	189.18±27.23	12.26±2.13	0.81±0.08	13.31±0.72	27.44±12.13	10.71±8.13	4.32±2.75
GG	N/A	N/A	N/A	N/A	N/A	N/A	N/A	N/A	N/A	N/A	N/A	N/A	N/A	N/A	N/A
P[a]	0.533	0.666	0.181*	0.182	0.525	0.572	0.413	0.145	0.899	0.027	0.403	0.082	0.198	0.955	0.443
**HOTAIR rs920778**															
TT	20.91±2.87	6.53±1.31	14.82±8.19	242.20±63.76	29.58±3.58	19.07±9.98	10.72±2.37	4.14±1.08	188.51±45.75	10.42±2.93	0.79±0.10	12.43±1.48	37.83±23.07	8.92±5.04	4.92±2.35
TC	20.85±2.39	7.17±1.70	16.26±12.94	233.86±53.68	29.14±3.30	19.15±8.42	10.85±2.23	3.77±0.79	186.52±40.69	10.58±2.86	0.78±0.10	12.71±1.35	40.86±32.45	9.34±5.41	4.81±2.56
CC	22.13±4.78	8.12±0.00	28.29±0.00	207.60±30.57	28.22±4.04	12.99±5.53	11.22±0.49	3.40±0.57	184.00±30.31	9.88±1.89	0.76±0.05	13.00±1.65	27.48±15.55	6.06±0.97	4.28±1.62
P[a]	0.487	0.193	0.405	0.389	0.603	0.199	0.867	0.244	0.956	0.863	0.826	0.508	0.506	0.343	0.818
**HOTAIR rs1899663**															
GG	20.97±2.79	6.52±1.29	15.98±10.79	240.84±63.68	29.73±3.77	19.32±9.74	10.90±1.97	4.34±1.09	187.73±43.61	10.64±2.95	0.79±0.10	12.54±1.45	37.39±22.13	8.77±4.87	4.88±2.34
GT	20.74±2.47	7.44±1.74	14.65±8.85	233.97±50.45	28.69±2.78	18.54±8.35	10.51±2.90	3.96±0.72	186.06±43.65	10.12±2.74	0.78±0.10	12.57±1.38	41.05±35.07	8.72±4.11	4.99±2.50
TT	22.26±5.53	N/A	N/A	206.00±46.13	27.70±0.66	1.07±4.38	10.80±0.76	3.50±0.71	203.00±33.18	9.80±1.06	0.80±0.10	12.90±2.26	31.58±6.98	11.73±11.77	3.34±2.02
P[a]	0.475	0.031	0.656	0.543	0.222	0.109	0.678	0.743	0.810	0.601	0.871	0.914	0.632	0.842	0.426
**HOTAIR rs7958904**															
GG	20.88±2.82	6.54±1.28	16.63±11.19	245.49±62.17	29.62±3.59	19.47±9.87	10.89±2.00	4.03±1.06	188.90±44.32	10.42±2.94	0.79±0.10	12.48±1.45	38.93±26.86	9.14±5.45	4.97±2.51
GC	20.72±2.38	7.52±1.87	11.95±5.52	233.42±55.35	28.91±3.18	18.35±8.34	10.77±2.42	3.89±0.84	185.29±42.98	10.62±2.83	0.78±0.10	12.57±1.45	39.20±26.71	8.83±4.42	4.63±2.15
CC	23.07±4.54	7.10±1.57	19.68±12.18	217.83±12.89	28.64±4.15	12.88±6.00	9.10±4.55	4.20±0.57	184.00±30.31	9.92±1.88	0.78±0.04	13.66±0.67	26.08±14.87	6.27±1.18	4.36±1.68
P[a]	0.092	0.099	0.286	0.122	0.526	0.153	0.232	0.847	0.904	0.858	0.737	0.206	0.508	0.413	0.743

Note:BMI,body mass index; PLT, platelet count; aPTT, Activated Partial Thromboplastin Time; PT, prothrombin time; T.chol, Total cholesterol; BUN, blood urea nitrogen; Hgb, hemoglobin

Supplementary Table 6. Differences of various clinical parameters according to HOTAIR gene polymorphisms in control

Genotypes	BMI (kg/m²) Mean ± SD (86)	Homocysteine (mmol/L) Mean ± SD (76)	Folate (mg/ml) Mean ± SD (8)	PLT (10³/μℓ) Mean ± SD (175)	aPTT (sec) Mean ± SD (67)	PT (sec) Mean ± SD (37)	Uric acid (mg/dl) Mean ± SD (7)	T.chol (mg/dl) Mean ± SD (9)	BUN (mg/dl) Mean ± SD (30)	Creatinine (mg/dl) Mean ± SD (30)	Hgb (mg/dl) Mean ± SD (171)	Estradiol (pg/ml) Mean ± SD (109)	FSH (mIU/ml) Mean ± SD (109)	LH (mIU/ml) Mean ± SD (106)
**HOTAIR rs4759314**														
AA	21.86±3.47	3.90±5.11	13.67±9.26	243.31±59.20	30.05±4.49	11.44±3.47	3.57±1.11	229.44±90.57	8.07±1.86	0.68±0.08	36.20±3.98	26.30±14.97	8.13±2.91	3.35±1.74
AG	20.66±2.43	2.59±1.66	N/A	234.39±78.94	32.32±4.98	10.93±0.38	N/A	N/A	7.90±0.00	0.70±0.00	35.72±4.56	25.93±11.12	8.64±1.97	2.91±2.14
GG	22.94±4.76	2.00±0.00	N/A	232.50±7.78	30.35±1.63	8.93±1.59	N/A	N/A	N/A	N/A	34.20±3.96	N/A	N/A	N/A
P^a	0.600	0.680	N/A	0.854	0.504	0.554	N/A	N/A	0.931	0.806	0.726	0.949	0.648	0.530
**HOTAIR rs920778**														
TT	21.73±3.25	3.29±3.28	16.91±10.73	239.28±54.86	30.18±4.63	10.81±0.96	3.30±1.09	183.67±42.30	8.09±1.71	0.69±0.08	36.16±4.19	26.07±14.48	8.30±3.00	3.76±1.94
TC	22.28±3.93	5.47±8.30	8.32±0.28	248.08±67.46	30.58±4.40	12.40±6.00	3.60±1.56	276.50±84.15	8.09±2.33	0.66±0.07	36.22±3.69	26.58±15.49	7.95±2.71	2.77±1.35
CC	20.73±3.35	2.63±1.77	8.19±0.00	239.86±74.61	29.78±4.05	10.82±0.77	4.60±0.00	410.00±0.00	7.20±0.00	0.60±0.00	35.13±4.57	25.35±3.32	8.90±2.26	2.90±0.71
P^a	0.606	0.216	0.515	0.654	0.928	0.415	0.665	**0.015**	0.899	0.438	0.791	0.981	0.772	**0.015**
**HOTAIR rs1899663**														
GG	21.64±3.35	3.16±3.20	10.78±3.97	237.89±56.67	30.18±4.59	10.76±0.85	2.60±0.57	174.60±40.25	7.88±1.55	0.68±0.08	36.16±4.15	26.57±14.63	8.29±2.98	3.58±2.03
GT	22.19±3.59	4.97±7.41	10.40±3.89	248.58±66.51	30.52±4.43	12.13±5.26	3.96±1.05	298.00±91.85	8.10±2.19	0.67±0.08	35.97±4.06	27.01±15.59	7.94±2.81	3.11±1.50
TT	20.98±4.01	4.20±3.11	35.05	240.90±44.42	27.70	N/A	N/A	N/A	10.90±0.00	0.80±0.00	37.17±1.97	21.25±10.59	8.57±2.48	3.02±1.30
P^a	0.754	0.336	**0.006**	0.528	0.815	0.215	0.154	**0.029**	0.283	0.324	0.677	0.525	0.741	0.364
**HOTAIR rs7958904**														
GG	21.96±3.37	3.36±3.35	11.54±3.97	241.63±55.35	29.56±4.39	10.70±0.94	3.65±1.26	216.00±93.86	7.79±1.52	0.68±0.08	36.26±3.94	26.43±14.78	8.22±3.03	3.79±2.02
GC	21.65±3.64	4.57±7.25	8.32±0.28	244.65±68.76	31.43±4.61	11.98±4.70	3.47±1.12	276.50±84.15	8.36±2.32	0.68±0.08	35.90±4.32	27.15±15.75	8.03±2.85	2.80±1.33
CC	20.37±2.42	3.10±2.20	35.05	237.600±46.48	29.60±2.69	10.05±0.00	N/A	N/A	10.90±0.00	0.80±0.00	36.54±2.84	22.50±10.30	8.45±2.28	3.47±1.73
P^a	0.650	0.594	0.247*	0.902	0.262	0.466	0.850	0.442	0.218	0.327	0.789	0.602	0.878	**0.022**

Note:BMI,body mass index; PLT, platelet count; aPTT, Activated Partial Thromboplastin Time; PT, prothrombin time; T.chol, Total cholesterol; BUN, blood urea nitrogen; Hgb, hemoglobin
*kruskal-wallis test
